# Fitness effects of synthetic and natural diet preservatives on the edible insect *Bombyx mori*

**DOI:** 10.1038/s41538-024-00284-9

**Published:** 2024-06-22

**Authors:** Xiaoyu Lei, Zhaoyi Qian, Xinyue Zhu, Nan Zhang, Jintao He, Jian Xiao, Xiaoqiang Shen, Abrar Muhammad, Chao Sun, Yongqi Shao

**Affiliations:** 1 Max Planck Partner Group, Institute of Sericulture and Apiculture, College of Animal Sciences, Zhejiang University, Hangzhou, China; 2 Analysis Center of Agrobiology and Environmental Sciences, Zhejiang University, Hangzhou, China; 3 Key Laboratory of Silkworm and Bee Resource Utilization and Innovation of Zhejiang Province, Hangzhou, China; 4 Key Laboratory for Molecular Animal Nutrition, Ministry of Education, Hangzhou, China

**Keywords:** Animal physiology, Antibiotics

## Abstract

Silkworm pupae as widely consumed insect products are good biosources of protein and micronutrients. Silkworm rearing throughout the year can be achieved by feeding them an artificial diet instead of native plants, facilitating extensive pupa production. However, artificial diets are prone to spoilage caused by bacterial contamination. Here, we evaluated the antiseptic effect of ethylparaben (EP, chemical preservative) and medium-chain fatty acids (MCFA, natural preservative) in a silkworm artificial diet. Results showed that both preservatives effectively inhibited pathogenic bacterial growth. Furthermore, the addition of EP or MCFA did not negatively impact the production capacity of silkworms and the homeostasis of gut microbiota. However, the expression of genes involved in detoxification such as *Ugt2*, and immune response such as *Cecropin B*, were upregulated after EP consumption. Therefore, natural preservative MCFA emerges as a suitable option from a safety perspective. These findings highlight future directions for improving insect artificial diet formulation.

## Introduction

**Fig. 1:**
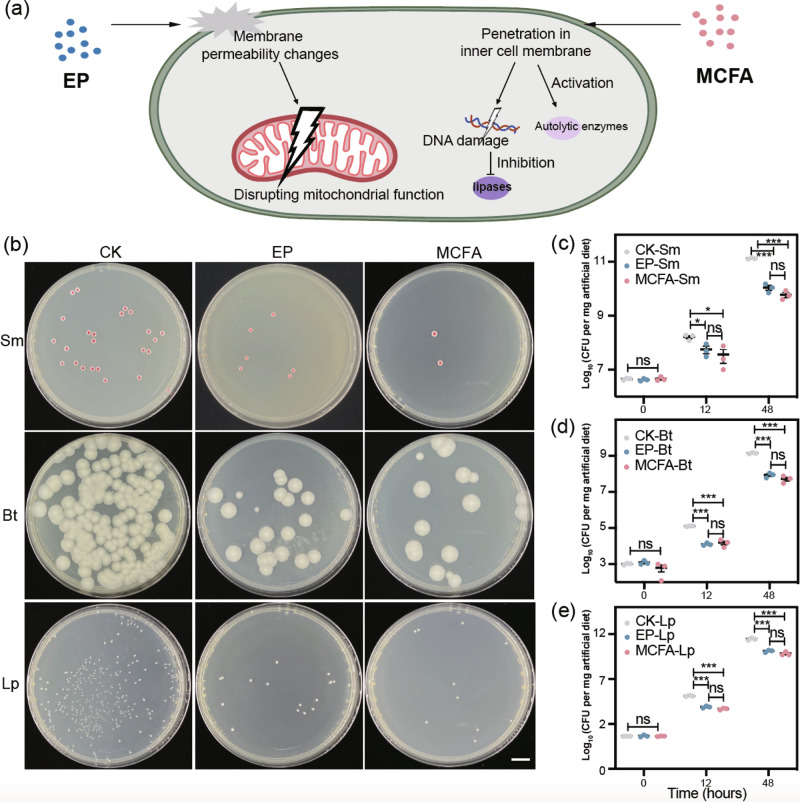
Antimicrobial efficacy of preservatives in artificial diet. **a** The mode of action of EP and MCFA. **b** Compared with CK, both EP and MCFA inhibited the growth of Sm, Bt, and Lp in artificial diet. Scale bar, 1 cm. **c**–**e** Changes in the CFU of Sm, Bt, and Lp in artificial diet after 12 and 48 h, indicating a significant inhibition of their growth by EP or MCFA, error bars indicate standard error of the mean (s.e.m.) (one-way ANOVA with Tukey’s *post hoc* test; *p < 0.05; **p < 0.01; ***p < 0.001; ns, not significant).

The silkworm (*Bombyx mori*) not only effectively produces silk, but the pupa also is a nutritionally rich bioresource^1–3^ and it can be utilized as dietary supplements, pharmaceuticals and healthcare products^4^. Silkworm pupa contains 71.9% total protein, 20.1% lipids, and 4.0% ash by dry weight. It also contains 18 essential amino acids, which meet the recommended amino acid pattern set by the Food and Agriculture Organization (FAO) and the World Health Organization (WHO)^5,6^. Furthermore, silkworm pupa includes many bioactive components with potential antioxidant, antibacterial, and therapeutic effects^7^. In China, silkworm pupae have been used as food for over 2000 years^8^. This tradition extends beyond China and enjoys popularity in various other Asian countries such as India, Thailand, Korea, and Japan^9,10^. In addition, silkworm pupae are also used as animal feed^11^. Several studies have shown that replacing up to 100% (w/w) of fish or soy meal with silkworm pupae in fish, pig, and poultry diets resulted in comparable growth rates, health, and nutritional value^5,12^. Currently, limited mulberry leaves and labor shortages constrain traditional sericulture. An urgent need exists for an “artificial diet” to enable continuous silkworm rearing and large-scale production of pupae^13^. Silkworm’s artificial diet is high in nutrients and moisture content^14^. While the high-temperature and humid atmosphere creates a conducive environment for silkworm feeding and growth, they also foster the proliferation of harmful microorganisms such as bacteria and mold^15^. Consequently, breeding silkworms on an artificial diet in non-sterile conditions leads to rapid diet spoilage and moldy, rotten feces, adversely affecting breeding. To maintain feed quality and prevent spoilage, adding suitable preservatives to the artificial feed is recommended^16^. Preservatives are substances capable of suppressing microbial activity and growth, thereby extending the shelf life of food and feed^17^. Ethylparaben (EP) is a type of paraben, which is chemically generated by the esterification of parahydroxybenzoic acid with alcohols. Paraben biocides generally act by disrupting mitochondrial function through induced membrane permeability changes and subsequent mitochondrial activation, leading to the loss of cellular adenosine triphosphate due to the disruption of oxidative phosphorylation (Fig. 1a)^16^. It has demonstrated remarkable efficacy as an antimicrobial agent and is used extensively, either individually or in combination with other compounds, as a preservative in foods, cosmetics, and drugs^18^.

In recent years, consumers are looking for their food with fewer artificial ingredients and processing steps^19^. Particularly, there has been a growing interest in the use of natural products as potential replacements for chemical preservatives, primarily due to lower toxicity^20^. For instance, bacteriocins and organic acids showed good antimicrobial activities against spoilage bacteria with minimal mammalian toxicity^21^. Medium-chain fatty acids (MCFA) correspond to saturated 6–12 carbon fatty acids and are naturally abundant in the form of medium-chain triglycerides (MCTs) found in sources like milk fat and various feed materials, principally coconut, palm, and cuphea seed oils^22^. MCFA possess antimicrobial properties and can be used for conserving feed and food^23^. In numerous in vitro experiments, MCFA and related monoglycerides have demonstrated their ability to inactivate harmful bacteria, viruses, and parasites. MCFA-induced antimicrobial effects can be attributed to their property as anionic surfactants^24^. The primary mechanisms potentially involved in the action of MCFA include membrane destabilization, which occurs through their absorption into the bacterial cell wall and cytoplasmic membrane, as well as the inhibition of bacterial lipases, which are necessary for cutaneous and intestinal mucosal colonization (Fig. 1a)^25^. In addition to the direct lytic actions of MCFA, the activation of bacterial autolytic enzymes may also contribute^26^.

Given the antiseptic properties of EP and MCFA, this study evaluated their efficacy in maintaining aseptic conditions in a silkworm artificial diet, as well as their potential impact on the fitness and overall health of silkworms, aiming to enhance the insect product’s safety. We found that adding the EP or MCFA did not negatively impact the production capacity of silkworms and the homeostasis of gut microbiota. However, EP as a chemical synthetic substance, exhibits a certain degree of toxicity, reflected in the upregulation of detoxification and immune-related genes. Therefore, MCFA presents a more suitable preservative from the safety perspective. A thorough understanding of these outcomes can help optimize silkworm-rearing practices, thereby sustainably producing this valuable insect food.

## Results and discussion

### EP and MCFA inhibit the growth of pathogenic bacteria

We first evaluated the antibacterial effect of EP and MCFA against three common silkworm pathogens. A preservative concentration of 0.1% exhibited the most effective antibacterial activity against the three silkworm pathogens (Supplementary Figure 1). Consequently, this concentration was selected for subsequent experiments. Following a 24 h incubation at 37 °C, both EP and MCFA demonstrated significant inhibition of the growth of *Serratia marcescens* (Sm), *Bacillus thuringiensis* (Bt), and *Lactobacillus plantarum* (Lp) on agar plates compared to the control (CK) (Supplementary Figure 1).

To further evaluate the antimicrobial properties of EP and MCFA within artificial diet, bacterial proliferation was monitored after inoculation into artificial diets amended with the two preservatives. Compared with CK, both EP and MCFA inhibited the growth of Sm, Bt, and Lp in the artificial diet too (Fig. 1b). Initially, there were no differences in bacterial colony forming units (CFU) observed among the three groups (Fig. 1c–e). However, significant inhibition of pathogen growth was observed at both 12 and 48 h post-inoculation in the artificial diets amended with either EP or MCFA, in comparison to CK. For instance, the CFU of Sm in the CK group was at least tenfold higher than those in the groups treated with EP and MCFA (Fig. 1c). The comparable trend was also observed for Bt and Lp (Fig. 1d, e).

### Impact of preservatives on silkworm economic traits

**Fig. 2:**
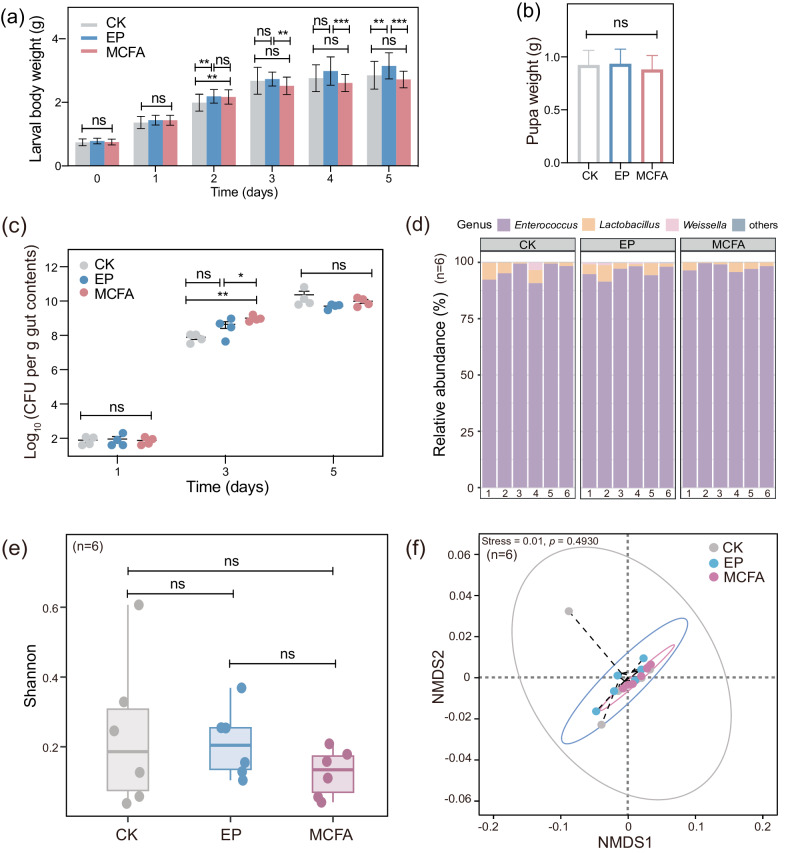
Changes in economic traits and gut microbiota of silkworms fed an artificial diet amended with EP or MCFA. **a** Adding the two preservatives into artificial diet had no negative effects on body weight. Error bars indicate standard deviation (s.d.). **b** Pupal weights did not differ among the three groups. Error bars indicate standard deviation (s.d.). The one-way ANOVA with Tukey’s post hoc test was used to assess the significant differences between the groups. *p < 0.05; **p < 0.01; ***p < 0.001; ns, not significant. **c** CFU counting of gut bacteria. Error bars indicate standard error of the mean (s.e.m). **d** The relative abundance of dominant bacterial taxa in the gut (*n* = 6). **e** Shannon index indicated no significant differences in species richness and community diversity, whether fed diets with or without preservatives (Wilcoxon rank-sum test, *p* > 0.05). **f** Nonmetric multidimensional scaling (NMDS) with Bray-Curtis showed that the bacterial communities of the CK, EP, and MCFA overlapped with each other and did not show a clear separation pattern (ANOSIM, *p* > 0.05). Each symbol represents a sample, colored by treatment.

We subsequently measured the silkworm body weight and pupa development when feeding EP or MCFA-amended artificial diets. Data analysis revealed that the silkworm body weight in the EP group gradually increased and was significantly higher on the 5th day as compared to the CK group, *p* < 0.05 (Fig. 2a). The group supplemented with MCFA and the CK group exhibited similar trends (Fig. 2a). It is noteworthy that pupal weights were consistent across all three groups (Fig. 2b). These results indicated that adding the two preservatives into artificial diet had no negative effects on important silkworm economic traits, including body weight and pupal weight.

### Response of the gut microbiota to dietary preservatives

To understand the possible influence of dietary preservatives on the host’s gut microbiota, analyses of gut bacterial load and composition were conducted following the consumption of artificial diets containing EP or MCFA. On the first day, there were no significant differences in CFU among the groups (Fig. 2c); on the third day, both EP and MCFA groups exhibited higher CFU compared to the CK group. After the application of the preservative, silkworms did not promptly adapt to it. This lack of adaptation may disrupt the homeostasis of the gut microbiota, consequently resulting in a higher CFU count after exposure to EP or MCFA compared to the CK group. However, by the fifth day, the differences became not significant, suggesting that the preservative-fed groups restored the homeostasis of gut microbiota to the same level as CK (Fig. 2c).

Next, we performed 16S rRNA gene high-throughput sequencing for the gut content. A total of 52 Amplicon Sequence Variants (ASVs) were identified from all samples. At the genus level, *Enterococcus* dominated the gut microbiota across all groups (95.96% in CK, 97.74% in EP, and 95.84% in MCFA) (Fig. 2d). The Shannon index revealed no significant differences in species richness or community diversity among fifth-instar silkworms consuming artificial diet either with or without preservatives (Fig. 2e). Similarly, no significant differences were found when other alpha diversity indices were assessed, including Chao 1, Pielou-e, and Simpson index (Wilcoxon rank-sum test, *p* > 0.05, Supplementary Figure 2). Nonmetric multidimensional scaling (NMDS) ordination based on Bray-Curtis showed that the bacterial communities of the CK (without preservative), EP, and MCFA overlapped with each other and did not show a clear separation pattern (Fig. 2f; ANOSIM, *p* > 0.05), suggesting that the addition of EP or MCFA did not cause significant changes in the composition and diversity of the silkworm gut microbiota.

### Host response to dietary preservatives

#### Transcriptomic analysis of differentially expressed genes (DEGs) and Gene Ontology (GO) enrichment

**Fig. 3:**
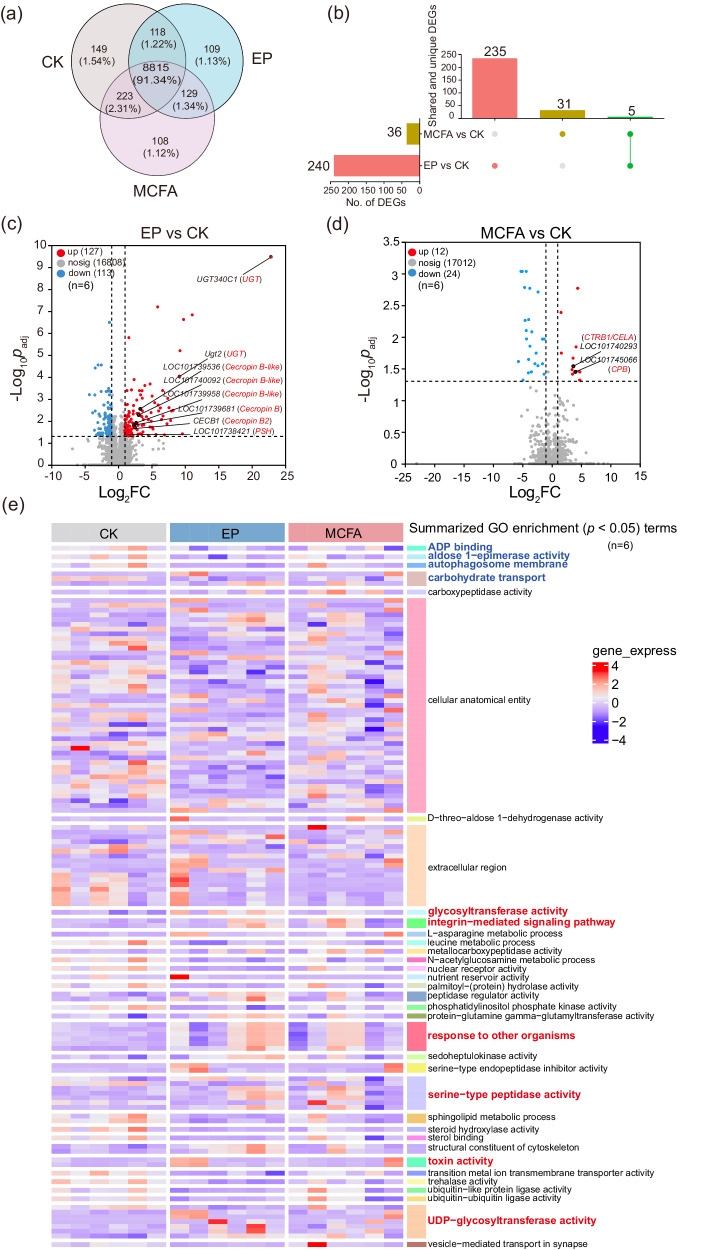
Gene expression changes of silkworms fed artificial diet amended with preservatives. **a** 8815 genes were shared by the CK and treatment groups, while 149, 109, and 108 genes were uniquely expressed in the CK, EP, and MPFAs groups, respectively. **b** The number of DEGs between different groups. **c**, **d** EP induced more substantial changes on silkworms, with higher numbers of DEGs (up = 127, down = 113) compared to MCFA (up = 12, down = 24). **e** The heatmap depicting the relative gene expression in significantly enriched GO terms (*p* < 0.05). The red and blue colors highlight up- and down-regulated genes.

To understand the effect of preservatives on the host, the transcriptome of the silkworm gut tissue was analyzed. Following the filtration of short and redundant reads, we obtained about 40–54 million clean reads (90.39–97.42%) from the CK, EP, and MCFA groups. Unigenes were annotated using six databases including GO, Kyoto Encyclopedia of Genes and Genomes (KEGG), Clusters of Orthologous Groups (COG), NCBI non-redundant protein sequences (NR), Swiss-Prot, and Protein families database (Pfam), which resulted in the identification of over 14,000 functional genes. Among these genes, a significant portion, 8815 genes (91.34%) were shared by both the CK and treatment groups, while 149, 109, and 108 genes were uniquely expressed in the CK, EP, and MCFA groups, respectively (Fig. 3a). Among the DEGs, 235 were unique to EP, 31 to MCFA, and five were common to both groups (Fig. 3b). Notably, four of the five shared genes lacked categorization in GO, KEGG, or other databases, and their functions remain unidentified. One gene associated with homeostasis, encoding nose resistance to fluoxetine protein 6, was downregulated. This gene plays a role in the absorption of various molecules from the gut to surrounding tissues. The down-regulation may serve as a protective response, reducing their absorption from the diet. Notably, EP had a more substantial effect on silkworms, with higher numbers of DEGs (up = 127, down = 113) compared to MCFA (up = 12, down = 24). Key genes of interest are highlighted in the figure and will be discussed later (Fig. 3c, d).

The GO classification, a universally accepted framework for the functional categorization of genes and gene products, was utilized to annotate DEGs^27^. Significantly, both the EP and MCFA groups were associated with enhanced catalytic activities within the molecular function domain (Supplementary Figure 3). In the EP group, an increased presence of membrane-associated DEGs in the cellular component category suggests potential effects on cell membrane structure. In contrast, the MCFA group was characterized by a greater number of extracellular region DEGs within the same category. The biological process category in the EP group revealed an abundance of terms associated with metabolic and cellular processes, indicating that EP may induce changes in metabolic pathways, with consequent implications for enzymatic activities in silkworms. Conversely, in the MCFA-fed group, only metabolic processes and localization-related DEGs were notably present in the cellular component category (Supplementary Figure 3). Analysis of the significantly enriched GO terms (*p* < 0.05) for genes in the EP and MCFA groups revealed distinctive patterns of expression. Genes with reduced expression were predominantly from families associated with ADP binding, aldose 1-epimerase activity, autophagosome membrane, and carbohydrate transport. Conversely, genes with enhanced expression were largely related to glycosyltransferase activity, integrin-mediated signaling pathways, responses to other organisms, serine-type peptidase activity, toxin activity, and UDP-glycosyltransferase activity (Fig. 3e).

#### KEGG enrichment analysis of DEGs

**Fig. 4:**
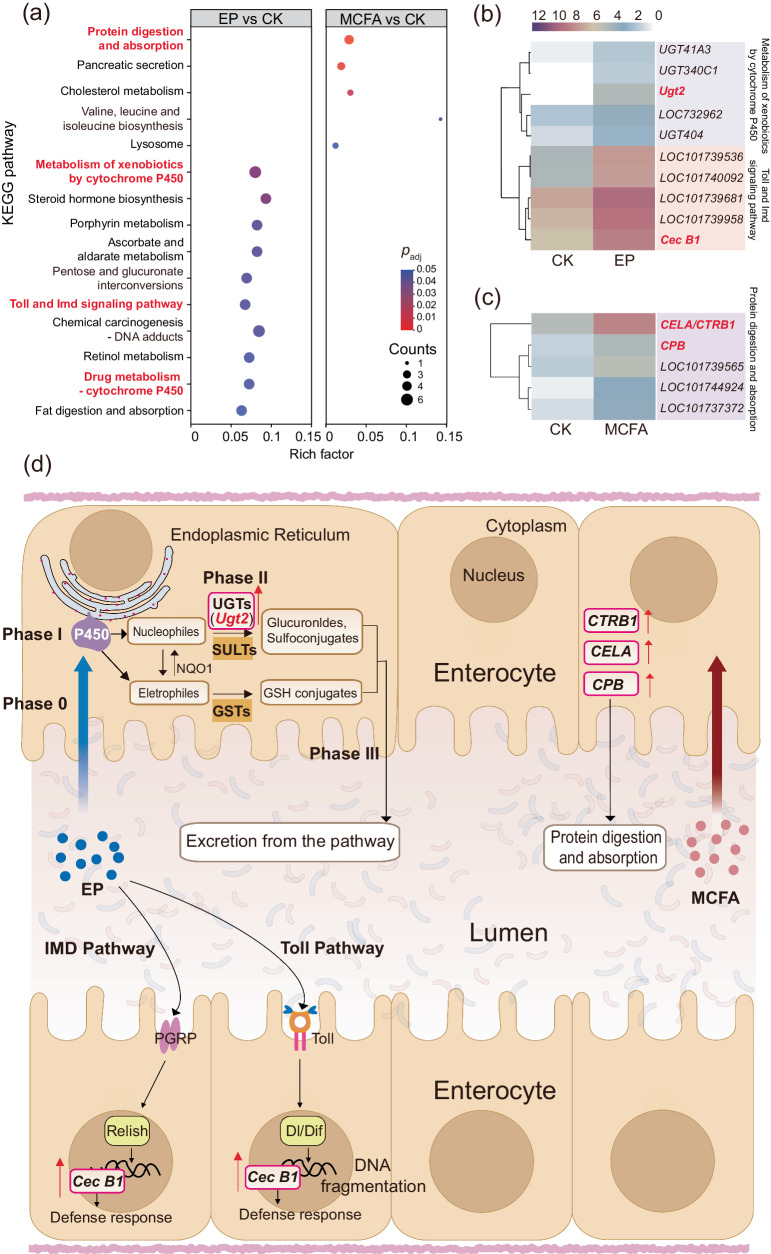
Pathway analysis and functional gene distribution and abundance clustering. **a** Enriched KEGG pathways under EP or MCFA treatment. **b**, **c** Clustering heatmap of genes involved in the metabolism of xenobiotics by cytochrome P450, Toll and IMD signaling pathway, and protein digestion and absorption pathway for EP (**b**) or MCFA (**c**) treatment. **d** EP-induced drug metabolism-cytochrome P450 pathways combined with UGTs, SULTs, and GSTs metabolic pathways, and the expression of the *Ugt2* gene in the UGTs metabolic pathway was upregulated. The *Cecropin B* (*Cec B1*) gene in the Toll and IMD pathway was upregulated, which may contribute to the enhanced defense response. The expression of *chymotrypsins* (*CTRB1*), *pancreatic elastase* (*CELA*), and *carboxypeptidase B* (*CPB*) involved in the pancreatic secretion pathway were upregulated after feeding on an artificial diet with MCFA, thereby promoting protein digestion and absorption.

Next, the analysis of the KEGG pathways provided further insights into the biological functions of the DEGs^28^. In the comparison between EP and CK, ten pathways were found to be significantly enriched (Fig. 4a), namely metabolism of xenobiotics by cytochrome P450, steroid hormone biosynthesis, porphyrin metabolism, ascorbate and aldarate metabolism, pentose, and glucuronate interconversions, Toll and IMD signaling pathway, chemical carcinogenesis-DNA adducts, retinol metabolism, drug metabolism-cytochrome P450, and fat digestion and absorption. Among them, we focused on the metabolism of xenobiotics by cytochrome P450, drug metabolism-cytochrome P450 and Toll and IMD signaling pathway. The metabolism of xenobiotics by cytochrome P450, and drug metabolism-cytochrome P450 were upregulated to varying degrees and responsible for the metabolic breakdown of drugs/xenobiotics via particular enzyme systems (e.g., the CYP450 family)^29^. Cytochromes P450 enzymes primarily function as oxidizing agents, with specific P450 families playing a pivotal role in drug metabolism^30^. According to recent investigations, parabens may serve as endocrine modulators or disruptors with mild estrogenic activity, which may have negative consequences on animals and human health^31^. Notably, paraben-containing antiperspirants have been linked with an increased risk of breast cancer, with non-metabolized parabens being found in small quantities in human breast cancers^32^. Cytochrome P450 often coordinates with other metabolic pathways such as UDP-glucuronosyltransferases (UGTs), sulfotransferases (SULTs), and glutathione S-transferases (GSTs) to jointly complete the detoxification-related metabolic functions. The expression of detoxification-related genes such as *Ugt2* in UGTs metabolic pathways was upregulated (Fig. 4b). When xenobiotics EP enter cells (often termed phase 0) (Fig. 4d), phase I enzymes such as cytochromes P-450, convert them into nucleophilic phenols and polyphenols or electrophiles like quinones and epoxides; these are then conjugated by phase II enzymes, such as UGTs and SULTs or GSTs, respectively; EP can cause upregulation of UGTS-related genes (*Ugt2*) in the detoxification pathway; the resulting organic anions are removed from the cell by export transporters (phase III)^33^. In addition, the *Cecropin B* (*Cec B1*) gene was upregulated in both the Toll and IMD pathways, a modification that is associated with an enhanced defense response^34^ (Fig. 4b, d).

In the comparison between MCFA and CK, we discovered five KEGG pathways enriched including protein digestion and absorption, pancreatic secretion, cholesterol metabolism, valine, leucine and isoleucine biosynthesis, and lysosome (Fig. 4a). Chymotrypsins (*CTRB1*), pancreatic elastase (*CELA*), and carboxypeptidase B (*CPB*) genes in protein digestion and absorption pathway were also upregulated (Fig. 4c, d). They may promote protein digestion and absorption^35^.

Collectively, when silkworm pupae are consumed as a food source, EP should be used cautiously as a preservative for silkworm artificial diet, and MCFA should be recommended instead. MCFA occurs naturally in milk fat and various feed materials, principally coconut, palm, and cuphea seed oils. And, in numerous Lepidoptera, Orthoptera, and Coleoptera, insufficient fatty acids in artificial diets have a deleterious influence on pupal eclosion, wing extension, and larval growth.

#### Gene set enrichment analysis (GSEA)

**Fig. 5:**
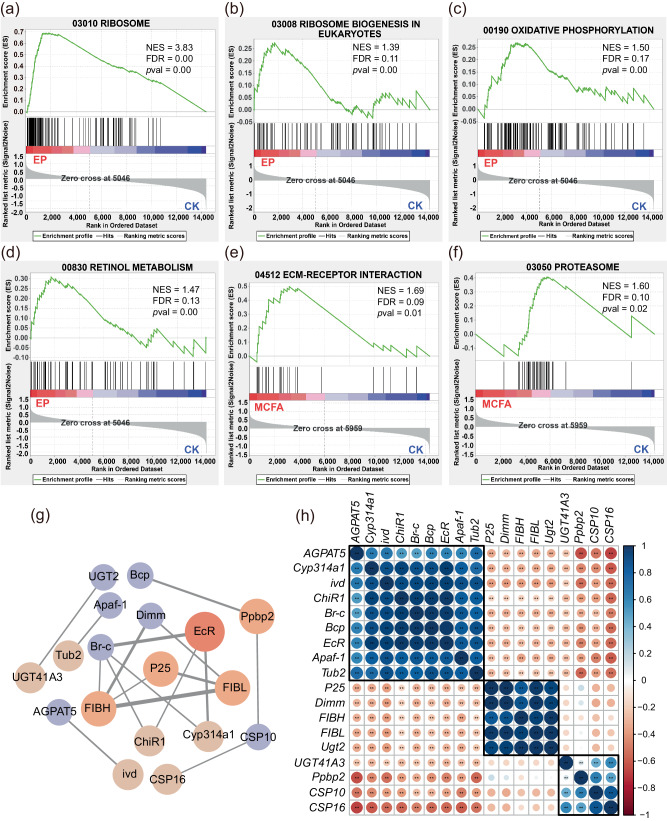
Gene set enrichment (GSEA) and PPI network analysis. **a**–**d** Compared with the CK group, the gene sets of metabolism of **a** ribosome, **b** ribosome biogenesis in eukaryotes, **c** oxidative phosphorylation, **d** retinol metabolism were significantly upregulated in EP-fed silkworms, while **e** ECM-receptor interaction, and **f** proteasome were significantly upregulated in MCFA-fed silkworms. Normalized Enrichment Score (NES), FDR q-value (FDR), and nominal *p*-value (*p*val) were determined by GSEA software and indicated within each enrichment plot. **g** PPI analysis of the DEGs/proteins was conducted in the STRING database (https://www.string-db.org/). Nodes represent genes, and edges represent the interaction between two genes (proteins), the thicker the edges, the stronger the interaction between the two proteins. The larger the node, the higher the gene score. The depth of color from red to violet indicates the rank of the protein interactions from high to low (by using the MCC method). **h** Co-expression analysis showed that the DEGs were closely co-expressed. **p < 0.01 based on Pearson correlation.

We used the GESA analysis to detect the genes with negligible expression changes but have substantial biological importance overall. Compared to the CK, there was a significant upregulation of the gene sets associated with the ribosome, ribosome biogenesis, oxidative phosphorylation, and retinol metabolism were significantly upregulated in the EP (Fig. 5a–d). The upregulation of the ribosome and ribosome biogenesis pathways, which are energy-intensive cellular processes, does not appear to be favorable for lifespan extension in various model organisms, including worms^36^. Enhanced activation of the oxidative phosphorylation pathway may induce oxidative stress and consequent damage in the testes of silkworms^37^. Retinol and its metabolites exert significant influences on the immune system, which include preserving the integrity of mucosal barriers and modulating the differentiation and functionality of immune cells^38^. In comparison with the control group (CK), there was a notable increase in both ECM (extracellular matrix)-receptor interaction and proteasome activity in the MCFA (Fig. 5e, f). Upregulation of ECM-receptor interaction primarily contributes to cellular adhesion, migration, differentiation, proliferation, and apoptosis^39^. Concurrently, the enhanced proteasome activity selectively degrades misfolded, damaged, or surplus proteins within the cell^40^. It is suggested that MCFA may facilitate these processes.

#### Protein-protein interaction (PPI) network analysis

Moreover, the PPI of DEGs associated with the two types of preservatives were examined using the STRING database. Overall, only 18 DEGs had interactions in EP-fed silkworms and 17 edges were found between the 18 DEGs, which were mainly involved in Apoptosis and UDP-glucoronosyl and UDP-glucosyl transferase metabolism pathway (Fig. 5g). Based on degree scores, CytoHubba identified five hub genes: ecdysone receptor (EcR), silk fibroin heavy chain (FIBH), silk fibroin light chain (FIBL), silk protein P25 (P25), and paralytic peptide binding protein 2 (Ppbp2) (Fig. 5g). Co-expression analysis further showed that the DEGs were closely co-expressed (Fig. 5h). In contrast, no interacting proteins were found in MCFA-fed silkworms.

### Validation of differentially expressed genes

**Fig. 6:**
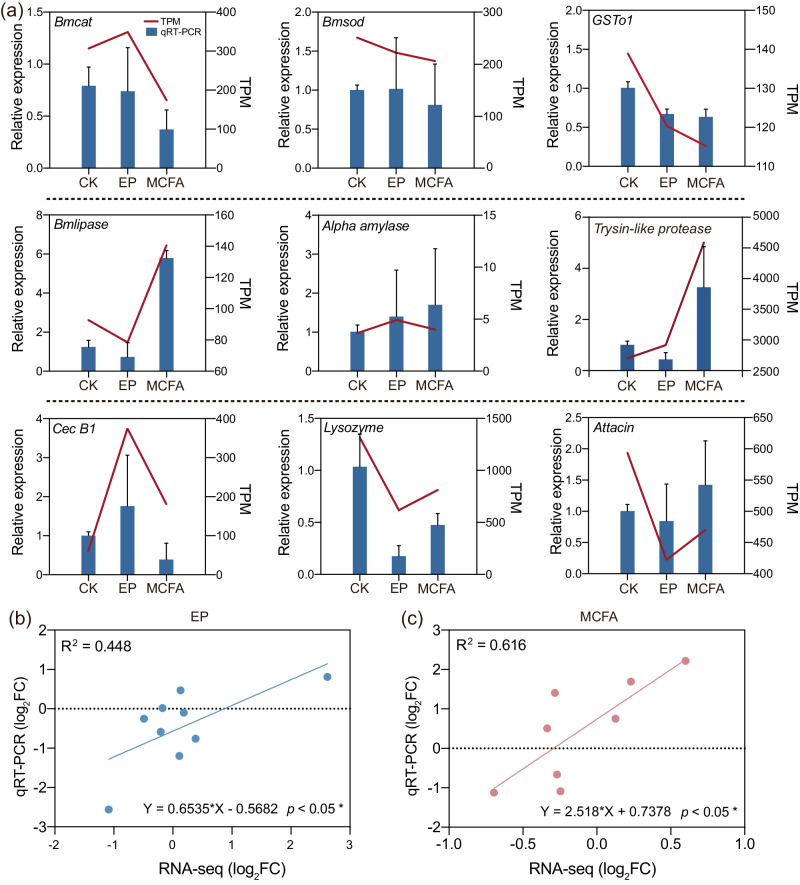
qRT-PCR validation of the key DEGs involved in host response to dietary preservatives. **a** The red lines represent TPM values in transcriptome data, and the blue column shown in the columns represents relative expression level by qRT-PCR. The expression of genes related to antioxidant activity (first line) was downregulated, indicating that preservative treatment may lead to weakened antioxidant capacity. Compared with the control group, the expression of digestive enzyme genes (second line) in the MCFA group was upregulated, MCFA may play a role in promoting digestion. Error bars indicate standard deviation (s.d.). **b**, **c** The correlation analysis used the expression changes (log_2_FC) of nine selected genes between qRT-PCR and RNA-Seq, indicating a significant positive correlation between qRT-PCR and RNA-Seq results (linear-fitting method (R^2^); *p < 0.05 based on Pearson correlation).

Finally, we selected nine key genes with diverse functions to validate the reliability of the RNA-Seq data. These genes were chosen specifically to investigate the impact of preservatives on silkworms, which are related to the antioxidant activity (*Bmcat*, *Bmsod*, *GSTo1*), digestive enzyme function (*Bmlipase, Alpha amylase, Trypsin-like protease*), and antimicrobial peptide activity (*Cecropin B*, *Lysozyme, Attacin*). As shown in Fig. 6a, the quantitative real-time PCR analysis (qRT-PCR) results were consistent with those of RNA-Seq. The correlation between changes in the expression of the selected genes based on RNA-Seq and qRT-PCR was analyzed by linear fitting (R^2^) and Pearson correlation methods, with R^2^ = 0.448 in EP group and R^2^ = 0.616 in MCFA group, and Pearson correlation coefficients (0.669 and 0.785) (Fig. 6b, c), indicating a significant positive correlation and consistency between the RNA-Seq and qRT-PCR results. Notably, the expression of genes related to antioxidant activity was downregulated, indicating that preservative treatment may lead to weakened antioxidant capacity. Compared with the CK, the expression of digestive enzyme genes in the MCFA group was upregulated, thus MCFA may play a role in promoting digestion. Simultaneously, we were attentive to the presence of large error bars in the qRT-PCR results, attributed to substantial inter-individual variations among silkworms, a recurring issue noted in prior studies too^41^.

In summary, adding preservatives to the artificial diet can suppress the growth of pathogens and does not exert a negative impact on the production capacity of silkworms and the homeostasis of gut microbiota. EP (Chemical preservative) is harmful to silkworms to a certain extent as indicated by the transcriptome data, which causes upregulation of *Ugt2* and *Cecropin B* in the detoxification and immune-related pathways. Meanwhile, natural MCFA demonstrated their suitability as a preservative by maintaining body weight and productivity, with the added advantage of being more biocompatible. Therefore, MCFA are more suitable as preservatives for the silkworm artificial diet, which deserve further study in other resource insects too.

## Methods

### Bacteriostatic test

The antibacterial effect of EP and MCFA was assessed against three microorganisms: the Gram-negative entomopathogen *Serratia marcescens* (Sm)^42^, the Gram-positive entomopathogen *Bacillus thuringiensis* (Bt)^43^, and *Lactobacillus plantarum* (Lp), a predominant food spoilage bacterium in the microbiota of silkworms fed on artificial diets, which adversely impacts host growth and development^44^. The bacteria were cultured in LB agar plates for 12–24 h at 37 °C. Single colonies were subsequently isolated for 16S rRNA gene identification and then subjected to secondary culture in LB broth. EP and MCFA were acquired from Rhawn and Hyatt zi (Shanghai) Biotechnology Co., LTD, respectively. Initially, we incorporated EP and MCFA into LB agar plates at various concentrations (0.05%, 0.1%, and 0.15%), and then uniformly inoculated them with 100 µL of each bacterial culture. LB agar plates devoid of preservatives served as the negative control (CK) for assessing the antibacterial efficacy of the two preservatives. The inoculated agar plates were incubated at 37 °C for 24 h.

### Evaluation of antimicrobial efficacy of preservatives in artificial diet

The artificial diet for silkworms, comprising 40% mulberry leaf powder, 30% soybean meal, 25% corn starch, 2.5% compound vitamins, and 2% inorganic salts, was hydrated with water in a 1:1.6 ratio and subsequently divided into three distinct groups. Sterile water was added to the control group (CK), while the treatment groups received either 0.1% EP or 0.1% MCFA. The mixtures were subsequently sterilized at 105 °C for 40 min. To evaluate the efficacy of EP or MCFA as preservatives in the artificial diet, it was inoculated with silkworm pathogens (Sm, Bt, and Lp). At 0, 12, and 48 h post-inoculation, identical volumes of the artificial diets from each of the three groups were plated onto LB agar plates and incubated at 37 °C for 24 h, followed by counting of CFU.

### Rearing silkworms on artificial diets amended with EP or MCFA

Normal fifth-instar silkworms (strain Jingsong×Haoyue) were randomly assigned into cohorts of thirty individuals (*n* = 30) each and were fed ad libitum on artificial diet: the control (CK), and those amended with EP or MCFA. Silkworms were maintained at a temperature of 25 °C and a relative humidity (RH) of 60%. Their body weight was recorded daily, and their diet was refreshed every 3 days. Besides, the pupal weight was also recorded.

### Gut bacterial load analysis and 16S rRNA gene sequencing

To quantify gut microbiota load, four silkworms (*n* = 4) from each group were dissected on the 1st, 3rd, and 5th days of the fifth instar to obtain gut contents. These contents were plated in equal volumes onto LB agar plates, incubated at 37 °C for 24 h, and CFU were subsequently counted.

For DNA extraction and gut microbiota analysis, gut contents were harvested from silkworms on the fifth day of the fifth instar, with each sample derived from an individual insect and six replicates (*n* = 6) prepared for each group. Genomic DNA was extracted from 20 mg of homogenized sample using the MasterPure™ Complete DNA and RNA Purification Kit (Epicentre, USA), according to the manufacturer’s instructions. The quality of bacterial DNA was assessed using a universal primer set, 27 F/1492R (Supplementary Table 1), targeting the bacterial 16S rRNA marker gene in a PCR assay. Subsequent PCR amplification products of the 16S rRNA gene V4 region with primers 515F/806R (Supplementary Table 1) were purified and quantified. These amplicons were then sequenced using the Illumina MiSeq platform (CA, USA). High-quality sequences were denoised using the q2-dada2 plugin with default parameters to differentiate the partial 16S rRNA gene ASVs and to remove chimeras^45^. Taxonomic assignment was executed utilizing a Naïve Bayes consensus taxonomy classifier within the SILVA database (version 138)^46^. ASVs identified as singletons, or those assigned to chloroplasts and mitochondria, were excluded^47^. Analyses of species richness, community diversity, and composition were conducted as described previously^48,49^. The R software (version 4.3.1) was used to compute the alpha and beta-diversity indexes of gut microbiota. Sequencing data have been deposited in the National Center for Biotechnology Information (NCBI) Sequence Read Archive under BioProject PRJNA1074245.

### Extraction of total RNA, library construction, and sequencing analysis

Host transcriptomic samples were collected concurrently with those for gut microbiome analysis. Individual silkworms, six replicates per group (*n* = 6), were anesthetized with 75% ethanol and subsequently rinsed with ddH_2_O. The entire gut was then dissected out using sterile fine forceps under aseptic conditions. RNA extraction was conducted using the Eastep^®^ Super Total RNA Extraction Kit (Promega, Shanghai, China), following the manufacturer’s guidelines. RNA quality was determined by 5300 Bioanalyzer (Agilent, USA) and quantified using the ND-2000 (NanoDrop Technologies, USA). Only high-quality RNA samples (OD260/280 = 1.8 ~ 2.2, OD260/230 ≥ 2.0, RIN ≥ 6.5, and 28S:18S ≥ 1.0) were used to construct the sequencing library. RNA purification, reverse transcription, library construction, and sequencing were performed at Shanghai Majorbio Bio-pharm Biotechnology Co., Ltd. (Shanghai, China) according to the manufacturer’s instructions (Illumina, San Diego, CA). Read quality assessment, transcriptome assembly, expression analysis, functional annotation, identification of DEGs, and functional enrichment analyses were executed as detailed in the prior study^50^. The abundance of transcript expression and gene expression levels were quantified by employing the Transcripts Per Million (TPM) method. Genes that exhibited an absolute log2 fold change (log_2_FC) greater than 1 and a false discovery rate (FDR) less than 0.05 were classified as DEGs. For differential expression analysis between the two groups, the DESeq2 package for R was utilized. Gene annotations included the KEGG pathway annotations and GO annotations. Figures were produced using the R software (version 4.3.1). The sequencing data have been deposited in the NCBI Sequence Read Archive under BioProject PRJNA1082399.

### Quantitative real-time PCR analysis (qRT-PCR)

Two µg of total RNA, identical to that used in the transcriptomic analysis, was utilized for cDNA synthesis employing the HiFair^®^ 1st Strand cDNA Synthesis Kit (Vazyme, Nanjing). The relative gene expression levels were quantified using a two-step qRT-PCR method, employing the 2^−ΔΔCt^ calculation. *α-Tubulin* served as the internal control, and cDNA was used as the template. The qPCR analysis targeted nine functional genes: *Bmcat*, *Bmsod*, *GSTo1*, *Bmlipase*, *Alpha amylase*, *Trypsin-like protease*, *Cecropin B*, *Lysozyme*, and *Attacin*^51^. Primers for these genes were designed with Primer Premier 6 software, and their sequences are provided in Supplementary Table 1.

### Statistical analysis

Differences in body weight, bacterial CFU, and gene expression changes among the CK and treatment groups were assessed using one-way ANOVA followed by Tukey’s post hoc test. The data were reported as mean values ± standard deviation. Unpaired comparisons between groups were analyzed using a Student’s *t*-test. A *p* value of <0.05 was considered significantly different (SPSS version 21.0, IBM, USA).

### Supplementary information

Supplementary information

## Data Availability

Sequencing data of 16S rRNA have been deposited in the NCBI Sequence Read Archive under BioProject PRJNA1074245. The sequencing data of transcriptome have been deposited in the NCBI Sequence Read Archive under BioProject PRJNA1082399.

## Code availability

All used codes are available on GitHub at https://github.com/Leixiaoyuuuuuu/Preservative/tree/master. This repository includes detailed documentation on the data processing and analysis methods used in this study.

## References

[1] Brogan EN, Park YL, Matak KE, Jaczynski J (2021). Characterization of protein in cricket (Acheta domesticus), locust (Locusta migratoria), and silk worm pupae (*Bombyx mori*) insect powders. LWT-Food Food Sci. Technol..

[2] Hazarika AK, Kalita U (2023). Human consumption of insects. Science.

[3] Malematja E, Manyelo TG, Sebola NA, Kolobe SD, Mabelebele M (2023). The accumulation of heavy metals in feeder insects and their impact on animal production. Sci. Total Environ..

[4] Sadat A (2022). Silkworm pupae as a future food with nutritional and medicinal benefits. Curr. Opin. Food Sci..

[5] Dai Y (2023). Enzymatic hydrolysis of silkworm pupa and its allergenicity evaluation by animal model with different immunization routes. Food Sci. Hum. Wellness.

[6] Tanga CM, Ekesi S (2024). Dietary and therapeutic benefits of edible insects: A global perspective. Annu. Rev. Entomol..

[7] Wu X, He K, Velickovic TC, Liu Z (2021). Nutritional, functional, and allergenic properties of silkworm pupae. Food Sci. Nutr..

[8] Marzoli F (2022). *Bombyx mori* from a food safety perspective: a systematic review. Food Res. Int..

[9] Mishra N, Hazarika N, Narain K, Mahanta J (2003). Nutritive value of non-mulberry and mulberry silkworm pupae and consumption pattern in Assam, India. Nutr. Res..

[10] Zhu L (2004). Exploitation and utilization of the silkworm Antheraea pernyi. North. Seric..

[11] Rangacharyulu PV (2003). Utilization of fermented silkworm pupae silage in feed for carps. Bioresour. Technol..

[12] Tassoni L (2022). Nutritional composition of *Bombyx mori* pupae: a systematic review. Insects.

[13] Bian D (2022). Evaluation of tolerance to λ-cyhalothrin and response of detoxification enzymes in silkworms reared on artificial diet. Ecotoxicol. Environ. Saf..

[14] Li J, Chen C, Zha X (2022). Midgut and head transcriptomic analysis of silkworms reveals the physiological effects of artificial diets. Insects.

[15] Zhou ZH (2008). Comparative proteomic analysis between the domesticated silkworm (*Bombyx mori*) reared on fresh mulberry leaves and on artificial diet. J. Proteome Res..

[16] Fransway AF (2019). Parabens. Dermatitis.

[17] Buckley HL (2017). Design and testing of safer, more effective preservatives for consumer products. ACS Sustain. Chem. Eng..

[18] Boberg J, Taxvig C, Christiansen S, Hass U (2010). Possible endocrine disrupting effects of parabens and their metabolites. Reprod. Toxicol..

[19] Erickson MC, Doyle MP (2017). The challenges of eliminating or substituting antimicrobial preservatives in foods. Annu. Rev. Food Sci. Technol..

[20] Ji J, Shankar S, Royon F, Salmieri S, Lacroix M (2023). Essential oils as natural antimicrobials applied in meat and meat products-a review. Crit. Rev. Food Sci. Nutr..

[21] Mokoena MP, Omatola CA, Olaniran AO (2021). Applications of lactic acid bacteria and their bacteriocins against food spoilage microorganisms and foodborne pathogens. Molecules.

[22] Dierick NA, Decuypere JA, Degeyter I (2003). The combined use of whole Cuphea seeds containing medium chain fatty acids and an exogenous lipase in piglet nutrition. Arch. Tierernahr..

[23] Woolford MK (1975). Microbiological screening of the straight chain fatty acids (C1‐C12) as potential silage additives. J. Sci. Food Agric..

[24] Mroz Z, Koopmans SJ, Bannink A, Partanen K, Radcliffe S (2006). Carboxylic acids as bioregulators and gut growth promoters in non-ruminants. Biol. Grow. Anim..

[25] Bergsson G, Steingrímsson O, Thormar H (2002). Bactericidal effects of fatty acids and monoglycerides on Helicobacter pylori. Int.J. Antimicrob. Agents.

[26] Tsuchido T, Hiraoka T, Takano M, Shibasaki I (1985). Involvement of autolysin in cellular lysis of Bacillus subtilis induced by short- and medium-chain fatty acids. J. Bacteriol..

[27] Zhao Y (2020). A literature review of gene function prediction by modeling gene ontology. Front. Genet..

[28] Kanehisa M, Goto S (2000). KEGG: kyoto encyclopedia of genes and genomes. Nucleic Acids Res..

[29] Zhai J, Man VH, Ji B, Cai L, Wang J (2023). Comparison and summary of in silico prediction tools for CYP450-mediated drug metabolism. Drug Discov. Today.

[30] Sun J (2022). Role of cytochrome P450 genes of Trichoderma atroviride T23 on the resistance and degradation of dichlorvos. Chemosphere.

[31] Liang J (2023). Studying paraben-induced estrogen receptor- and steroid hormone-related endocrine disruption effects via multi-level approaches. Sci. Total Environ..

[32] Iribarne-Durán LM (2022). Biomonitoring bisphenols, parabens, and benzophenones in breast milk from a human milk bank in Southern Spain. Sci. Total Environ..

[33] Bock KW (2003). Vertebrate UDP-glucuronosyltransferases: functional and evolutionary aspects. Biochem Pharm..

[34] Valanne S, Wang J-H, Rämet M (2011). The Drosophila toll signaling pathway. J. Immunol..

[35] Németh BZ (2022). Arg236 in human chymotrypsin B2 (CTRB2) is a key determinant of high enzyme activity, trypsinogen degradation capacity, and protection against pancreatitis. Biochim. Biophys. Acta Proteins Proteom..

[36] Xiao FH (2022). ETS1 acts as a regulator of human healthy aging via decreasing ribosomal activity. Sci. Adv..

[37] Tang W (2021). Sodium fluoride causes oxidative damage to silkworm (*Bombyx mori*) testis by affecting the oxidative phosphorylation pathway. Ecotoxicol. Environ. Saf..

[38] Gudas LJ (2022). Retinoid metabolism: new insights. J. Mol. Endocrinol..

[39] Hu C (2022). Retinoic acid promotes formation of chicken (Gallus gallus) spermatogonial stem cells by regulating the ECM-receptor interaction signaling pathway. Gene.

[40] Thibaudeau TA, Smith DM (2019). A practical review of proteasome pharmacology. Pharm. Rev..

[41] Xiang H (2018). The evolutionary road from wild moth to domestic silkworm. Nat. Ecol. Evol..

[42] Raymann K, Coon KL, Shaffer Z, Salisbury S, Moran NA (2018). Pathogenicity of Serratia marcescens Strains in Honey Bees. mBio.

[43] Ugur A, Gholamreza SJ, Estibaliz S, Vincent SB (2023). Biotechnological advances in Bacillus thuringiensis and its toxins: Recent updates. Rev. Environ. Sci. Bio..

[44] Orlo E, Russo C, Nugnes R, Lavorgna M, Isidori M (2021). Natural methoxyphenol compounds: antimicrobial activity against foodborne pathogens and food spoilage bacteria, and role in antioxidant processes. Foods.

[45] Callahan BJ (2016). DADA2: high-resolution sample inference from Illumina amplicon data. Nat. Methods.

[46] Bokulich NA (2018). Optimizing taxonomic classification of marker-gene amplicon sequences with QIIME 2’s q2-feature-classifier plugin. Microbiome.

[47] He J (2023). Primer selection impacts the evaluation of microecological patterns in environmental microbiomes. iMeta.

[48] Qiu J, Cheng Y, Deng Y, Ren G, Wang J (2023). Composition of gut microbiota involved in alleviation of dexamethasone-induced muscle atrophy by whey protein. NPJ Sci. Food.

[49] Shen Y (2023). Lipid complexation reduces rice starch digestibility and boosts short-chain fatty acid production via gut microbiota. NPJ Sci. Food.

[50] Vera-Ponce de León A, Jahnes BC, Otero-Bravo A, Sabree ZL (2021). Microbiota perturbation or elimination can inhibit normal development and elicit a starvation-like response in an omnivorous model invertebrate. mSystems.

[51] Muhammad A (2021). Toxic effects of acute exposure to polystyrene microplastics and nanoplastics on the model insect, silkworm *Bombyx mori*. Environ. Pollut..

