# Supplementary material for: Fitness effects of synthetic and natural diet preservatives on the edible insect Bombyx mori
Source: NPJ Sci Food. 2024 Jun 22;8:39. doi: 10.1038/s41538-024-00284-9 (PMC11193800; doi:10.1038/s41538-024-00284-9)
Supplement: Supplementary file 1 — Supplementary information [file 41538_2024_284_MOESM1_ESM.pdf]

**Supplementary materials**

Supplementary figures

**Supplementary Figure 1** Preservative concentration test, corresponding to text (Methods) Bacteriostatic test and (Result and discussion) EP and MCFA inhibit the growth of pathogenic bacteria.

**Supplementary Figure 2** Alpha diversity of the silkworms' gut bacterial community, corresponding to text (Result and discussion) Response of the gut microbiota to dietary preservatives.

**Supplementary Figure 3** GO classification of DEGs, corresponding to text (Result and discussion) Transcriptomic analysis of DEGs and GO enrichment.

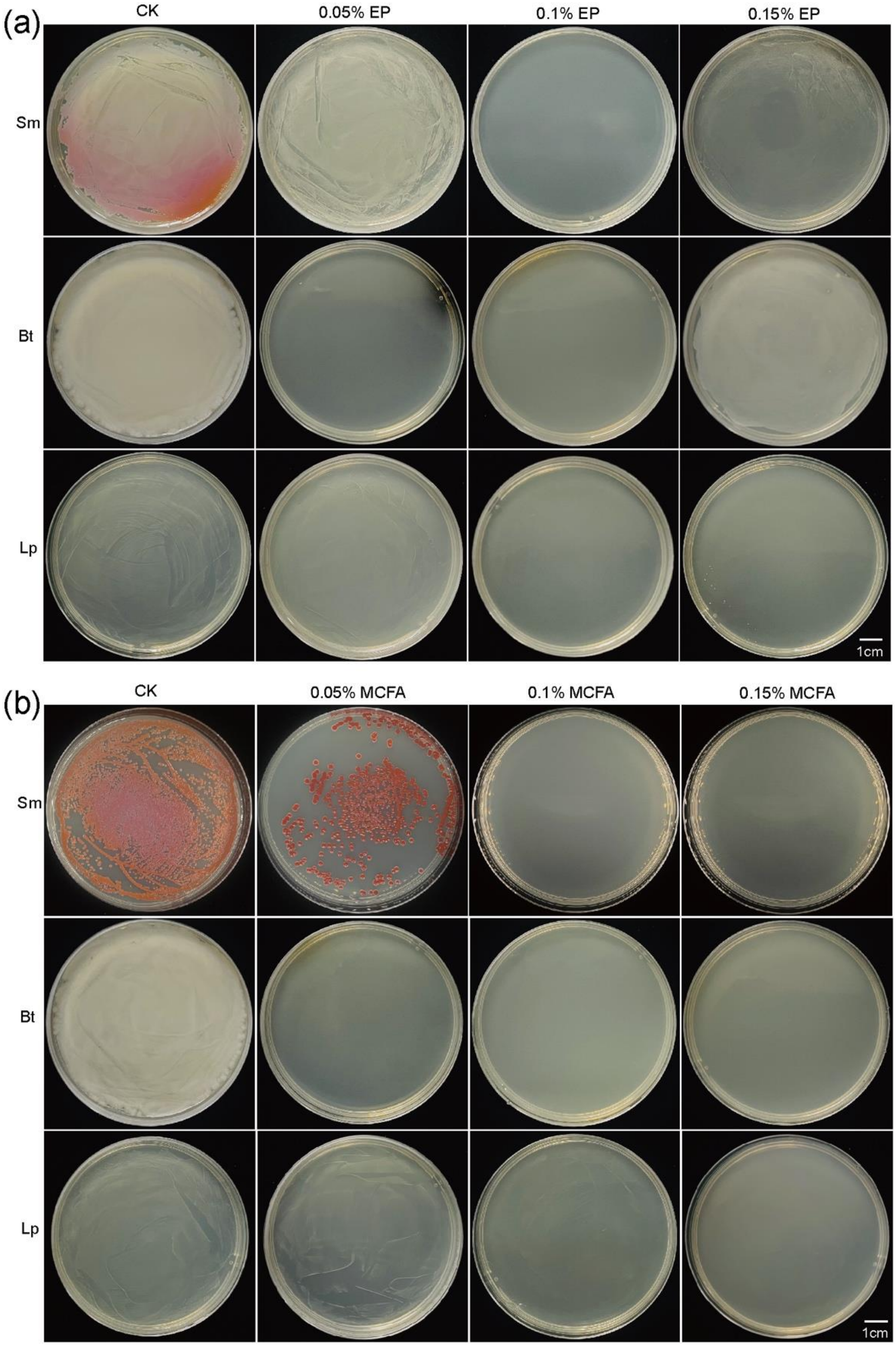


**Supplementary Figure 1** The preservative concentrations of 0.05%, 0.1%, and 0.15% were tested A volume of 100 μL of each three bacteria was inoculated on LB solid medium containing different concentrations of EP (a) and MCFA (b).

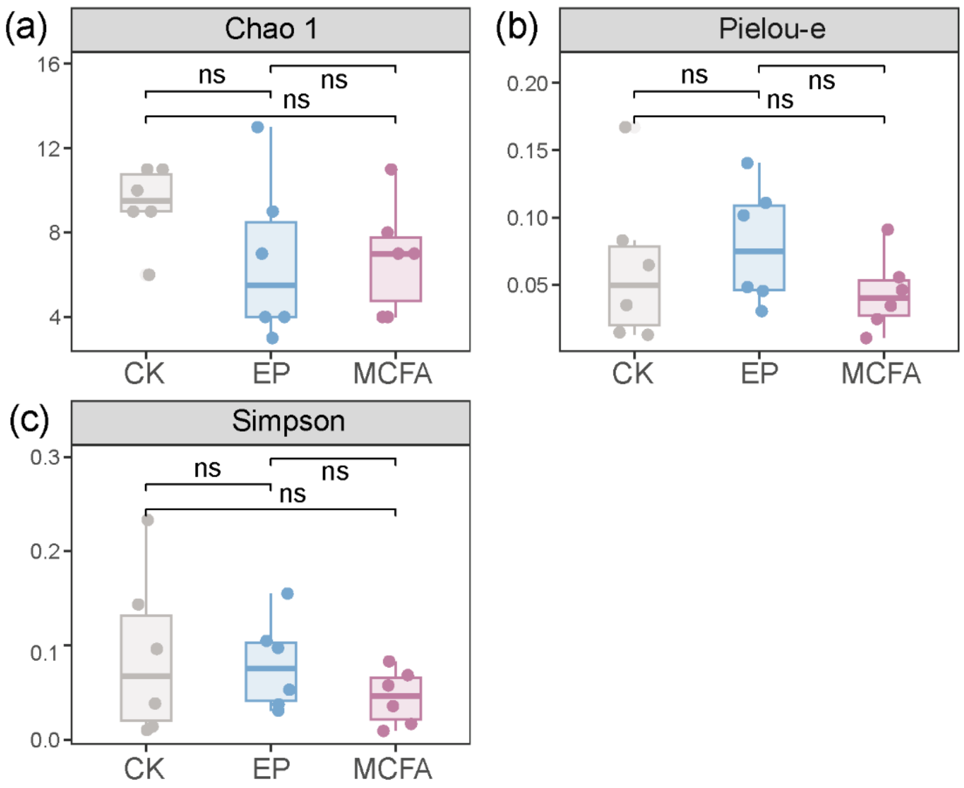


**Supplementary Figure 2** Alpha diversity of the silkworms' gut bacterial community. (a) The Chao index estimates the total number of species included in the community samples. (b) Pielou-e index reflects the evenness, and the larger the value, the more uniform. (c) Simpson index: to characterize the diversity and evenness of species distribution within a community. ASV richness and diversity values were calculated for genus-level ASVs. Data were analyzed using the Wilcoxon rank-sum test ($P > 0.05$).

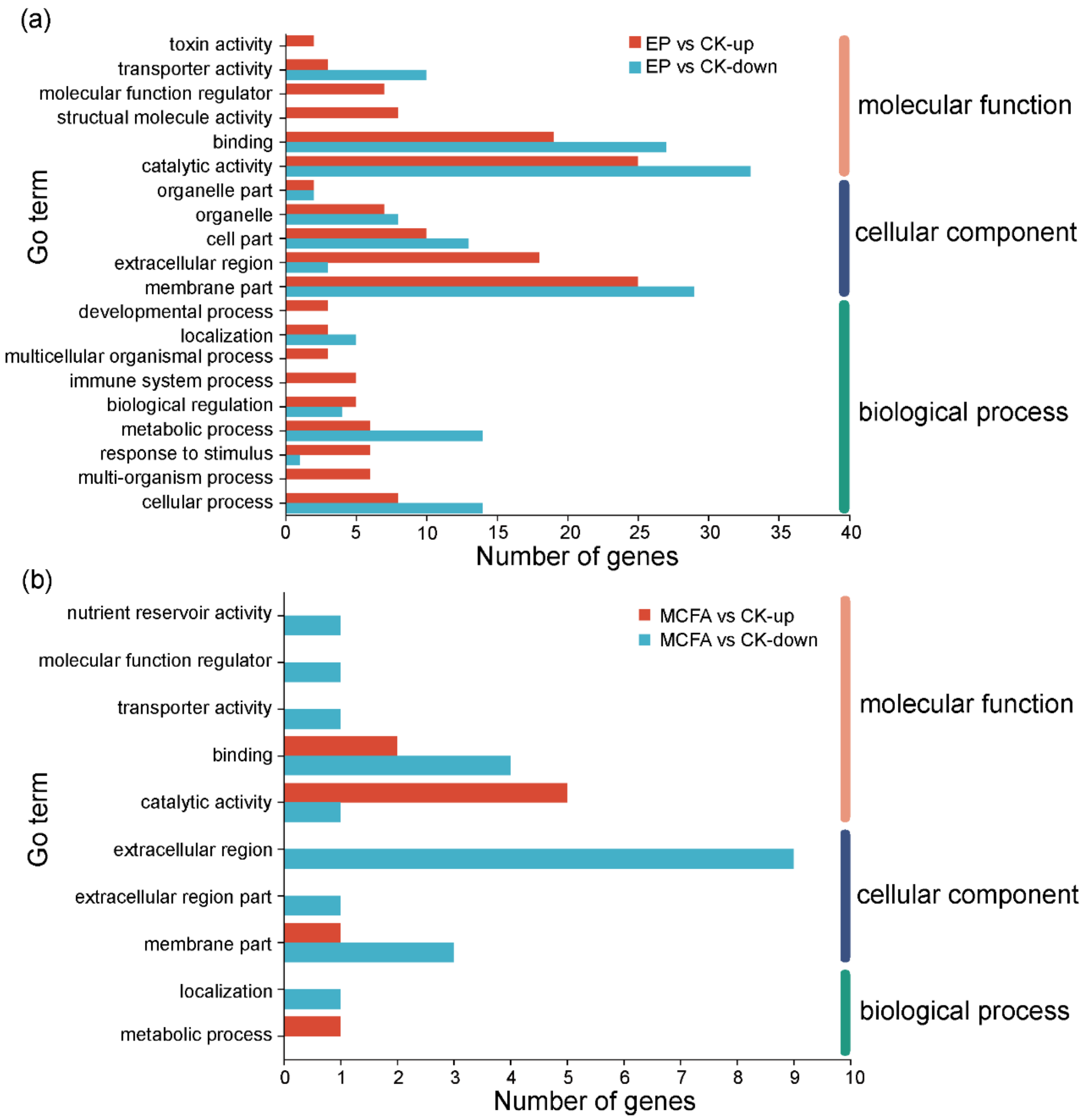


**Supplementary Figure 3** DEGs enrichment analysis between the CK and EP treatment group (a), and between the CK and MCFA treatment group (b) based on Gene Ontology.

Supplementary table

**Supplementary Table 1** All the primers used in this study, corresponding to text (Methods) Gut bacterial load analysis and 16S rRNA gene sequencing and Quantitative real-time PCR analysis (qRT-PCR).

| **Primer** | **Sequence (5′-3′)** | **Usage** |
|---|---|---|
| DB200F | CTGGAACAGCAACGGAAACT | forward primer for qPCR analysis gut bacteria quantification |
| DB200R | TCCGTTGTTGATGAGCCAGA | reverse primer for qPCR analysis gut bacteria quantification |
| 27F | AGAGTTTGATCCTGGCTCAG | forward primer for checking bacterial DNA quality in a PCR reaction |
| 1492R | TACGGYTACCTTGTTACGACTT | reverse primer for checking bacterial DNA quality in a PCR reaction |
| 515F | GTGYCAGCMGCCGCGGTAA | forward primer for PCR amplification of V4 hypervariable regions of eubacterial 16S rRNA gene |
| 806R | GGACTACHVGGGTWTCTAAT | reverse primer for PCR amplification of V4 hypervariable regions of eubacterial 16S rRNA gene |
| α-*tubulin*F | ACATGGCTTGCTGTATGCT | forward primer for qRT-PCR analysis of *α-tubulin* gene (as reference) |
| α-*tubulin*R | GGGTGGCTGGTAGTTGATA | reverse primer for qRT-PCR analysis of *α-tubulin* gene (as reference) |
| *Bmcat*F | TCCTACGGGAGGCAGCAGT | forward primer for qRT-PCR analysis of *Bmcat* gene |
| *Bmcat*R | GGACTACCAGGGTATCTAATCCTGTT | reverse primer for qRT-PCR analysis of *Bmcat* gene |
| *Bmsod*F | ACTCCTACGGGAGGCAGCA | forward primer for qRT-PCR analysis of *Bmsod* gene |
| *Bmsod*R | GGACTACHVGGGTWTCTAAT | reverse primer for qRT-PCR analysis of *Bmsod* gene |
| *GSTo1*F | CTTCCTCGTCGGAGCATTCT | forward primer for qRT-PCR analysis of *GSTo1* gene |
| *GSTo1*R | GTTCGCCTTAACTGCCATCAA | reverse primer for qRT-PCR analysis of *GSTo1* gene |
| *Bmlipase*F | AGAAATTCCAAACGAACTTG | forward primer for qRT-PCR analysis of *Bmlipase* gene |
| *Bmlipase*R | CAGTGCTCTACCTCCATCATT | reverse primer for qRT-PCR analysis of *Bmlipase* gene |
| *Alpha amylase*F | ACTTTAAGTTGGGAGGAAGGG | forward primer for qRT-PCR analysis of *Alpha amylase* gene |
| *Alpha amylase*R | ACACAGGAAATTCCACCACCC | reverse primer for qRT-PCR analysis of *Alpha amylase* gene |

| | | |
|---|---|---|
| *Trysin-like protease*F | GTCTATTTCGCACGTCGTGCTTTGC | forward primer for qRT-PCR analysis of *trysin-like protease* gene |
| *Trysin-like protease*R | CTTCTCAACTGCGCGGATGAGACC | reverse primer for qRT-PCR analysis of *trysin-like protease* gene |
| *Cecropin B*F | CCTATCCTTCGTCTTCGCTCT | forward primer for qRT-PCR analysis of *Cecropin B* gene |
| *Cecropin B*R | TAGCTTTAGCCGAACCAAGG | reverse primer for qRT-PCR analysis of *Cecropin B* gene |
| *Lysozyme*F | TTCTGAAGCCAAAACGTTCA | forward primer for qRT-PCR analysis of *Lysozyme* gene |
| *Lysozyme*R | TGGAACAATCCGTAGTCCTT | reverse primer for qRT-PCR analysis of *Lysozyme* gene |
| *Attacin*F | TTCAAACAGAAGGTGGGC | forward primer for qRT-PCR analysis of *Attacin* gene |
| *Attacin*R | GACGGAGATTGGAACAGG | reverse primer for qRT-PCR analysis of *Attacin* gene |

28
